# COVID-19 Vaccine Experience: Loss of Humoral Response Following Autologous Stem Cell Transplantation in Multiple Myeloma Patients and Positive Effect of Booster Dose

**DOI:** 10.3390/jcm14134648

**Published:** 2025-07-01

**Authors:** Uros Markovic, Elvira Scalisi, Giuliana Giunta, Antonella Nardo, Andrea Duminuco, Nunziatina Laura Parrinello, Sara Marino, Valeria Iachelli, Giulio Antonio Milone, Paola Scirè, Gabriella Amato, Federica Galbo, Giuseppe Milone, Emanuele Martorana, Alessandra Romano, Concetta Conticello, Francesco Di Raimondo, Gaetano Moschetti, Daniela Carcò

**Affiliations:** 1 Hematology Unit with BMT A.O.U. Policlinico “G. Rodolico-San Marco”, Via S. Sofia 78, 95123 Catania, Italy; scalisi.e97@gmail.com (E.S.); giulianagiunta24@gmail.com (G.G.); antonella.nardo@live.it (A.N.); andrea.duminuco@gmail.com (A.D.); laura.parrinello@tiscali.it (N.L.P.); saramarino92@virgilio.it (S.M.); giuseppe.milone@gmail.com (G.M.); sandrina.romano@gmail.com (A.R.); ettaconticello@gmail.com (C.C.); francesco.diraimondo@unict.it (F.D.R.); 2 Unità Operativa di Patologia Clinica PO Umberto I, Via Testaferrata 1, 99100 Siracusa, Italy; valeria.iachelli@gmail.com; 3 Mediterranean Institute of Oncology, Via Penninazzo 7, 95029 Viagrande, Italy; giulio.milone89@gmail.com (G.A.M.); paola.scire@grupposamed.com (P.S.); gabriella.amato@grupposamed.com (G.A.); federica.galbo@grupposamed.com (F.G.); emanuele@martorana.email (E.M.); gaetano.moschetti@grupposamed.com (G.M.); daniela.carco@grupposamed.com (D.C.)

**Keywords:** autologous stem cell transplantation, COVID-19 pandemic, multiple myeloma

## Abstract

**Background/Objectives**: This prospective study investigated the impact of high-dose chemotherapy and autologous stem cell transplantation (ASCT) on anti-COVID-19 antibody levels in previously vaccinated multiple myeloma (MM) patients with confirmed antibody response (AR). **Methods**: All patients underwent at least a two-dose regimen mRNA vaccination and later received a high-dose melphalan conditioning regimen and ASCT. **Results**: Fourteen MM patients with confirmed AR underwent a total of nineteen ASCT reinfusions; their median age was 55 (34–67). The study found a significant and progressive decrease in antibody levels after ASCT, from 311 BAU/mL at baseline to 276 BAU/mL and 188 BAU/mL after one and three months, respectively, with a median anti-COVID-19 antibody level reduction of 39% (range 16–66%) that was statistically significant (*p* = 0.014) using the Friedman test. However, the third “booster” vaccination post-ASCT improved the humoral response at six months in nine patients (50% response rate) and corrected, at least in part, the negative impact of high-dose chemotherapy (*p* = 0.597). Despite the antibody decline, three patients who contracted COVID-19 after ASCT experienced mild, outpatient-managed infections, suggesting sufficient immune response. Furthermore, booster doses increased the proportion of high-responders (AR > 500 BAU/mL) post-ASCT from 22% to 55% (5/9 patients) at three and six months, respectively. **Conclusions**: The study concludes that ASCT negatively affects the humoral response, but booster vaccination can improve it, and residual antibodies may prevent severe COVID-19 in these vulnerable patients.

## 1. Introduction

The Severe Acute Respiratory Syndrome Coronavirus 2 (SARS-CoV-2) pandemic has significantly influenced healthcare systems and everyday life since December 2019. Novel coronavirus disease 2019 (COVID-19) vaccines with innovative messenger RNA (mRNA) mechanisms, namely NT162b2 (Pfizer [New York City, NY, USA]/BioNTech [Mainz, Germany]) and mRNA-1273 (Moderna [Cambridge, MA, USA]), were approved by both the FDA and EMA and dramatically improved the outcome, allowing the end of the emergency phase. The approval was based on large phase III clinical trials with over 90% efficacy in preventing symptomatic SARS-CoV-2 disease following the programmed two-dose regimen and production of antibodies against the spike component of COVID-19 [1,2]. However, patients with hematological malignancies were not included in the registration trials due to immune depression caused by both the disease itself and the immunochemotherapy regimens. In Italy, mass prophylactic vaccination with mRNA vaccines was initiated in 2021 starting with healthcare workers, followed by vulnerable populations.

The regular vaccination schedule for hematological patients, according to disease stage and hematopoietic stem cell transplantation (HSCT), was based on the European Conference on Infections in Leukemia (ECIL) guidelines during the COVID-19 pandemic [3,4]. The vaccination’s ideal timing, if possible, was before anti-neoplastic treatment started, three to six months after chemotherapy, or during maintenance therapy with variable response efficacy. Furthermore, a re-vaccination program remained recommended for HSCT recipients following both allogeneic and autologous transplantation, due to weakened immune response.

As for the COVID-19 virus, several studies have demonstrated a high risk of severe infections in patients with hematological malignancies, with an over 30% mortality rate and prolonged virus persistence [5,6,7,8,9], including those that underwent HSCT [10,11,12]. In our patients, over time, we also demonstrated the role of vaccination in order to guarantee the best outcome and ensure the continuation of treatment without delays [13,14,15].

Therefore, oncological and hematological patients with active disease were included in the vulnerable group in Italy, even though a diagnosis of active malignancy represented an exclusion criterion in the pivotal trials [1,2]. Given the higher mortality risk and need for prolonged inpatient hospitalization with respiratory life support in many cases and based on international and national recommendations, COVID-19 mRNA vaccination was advised in all hematological patients. The timing was also possible between treatment cycles; even if the efficacy may not have been optimal, given that a minor immune response alone could determine a less-severe disease manifestation in this setting of patients [16,17,18]. Furthermore, ECIL guidelines confirmed the recommendation for vaccination boosters following autologous (ASCT) and allogeneic HSCT, given the extremely high probability of immunity loss.

We conducted a prospective study in consecutive hematological patients affected by multiple myeloma (MM), who had an immune response to the COVID-19 mRNA vaccination, that were regularly monitored in order to evaluate the impact of high-dose chemotherapy on the maintenance of humoral response following ASCT, along with the importance and timing of subsequent re-vaccination.

## 2. Materials and Methods

### 2.1. Study Design

The aim of our prospective study was to monitor the level of COVID-19 antibody immunity in previously vaccinated adult MM patients with confirmed humoral anti-COVID-19 antibody response (AR) (targeted antibody titer level against receptor-binding domain (RBD) of the spike protein S1 subunit greater than 17.8 BAU/mL) according to VITROS Immunodiagnostic Products Anti-SARS-CoV-2 IgG Quantitative test.

Inclusion criteria were as follows:

- Multiple myeloma patients eligible for ASCT according to general EBMT guidelines [19]
- Administration of two-dose regimen mRNA vaccination prior to ASCT (namely NT162b2 or mRNA-1273)
- Confirmed baseline humoral anti-COVID-19 AR with targeted antibody titer level greater than 17.8 BAU/mL

Exclusion criteria were:

- MM patients not eligible for ASCT
- Baseline humoral anti-COVID-19 antibody response less than 17.8 BAU/mL cut-off

All patients received a high-dose melphalan (HD-PAM) conditioning regimen and ASCT between January and December 2022. Patients that received the third vaccination “booster” dose or suffered from SARS-CoV-2 infections were also registered. Possible episodes of COVID-19 positivity/infection both prior to and following mRNA vaccination were also assessed.

Data were collected from the patient’s medical records and pre-scheduled follow-up visits, including laboratory exams, booster mRNA SARS-CoV-2 vaccine doses, and a potential COVID-19 infection update. The study was approved by an independent ethics committee (n.33/2022/C.E., 14 January 2022) and conducted in accordance with the International Conference on Harmonization Guidelines on Good Clinical Practice and the principles of the Declaration of Helsinki. All patients provided written informed consent.

The eligibility and indication for ASCT were assessed according to general EBMT guidelines [19]. A high-dose chemotherapy conditioning regimen with melphalan (HD-PAM) was used, while possible episodes of COVID-19 positivity/infection both prior to and following mRNA vaccination were also assessed.

Antibody titer against S1 subunit Spike protein RBD was evaluated using the VITROS Anti-SARS-CoV-2 IgG Quantitative test. The World Health Organization established an international standard and reference panel for anti-SARS-CoV-2 antibodies, which was aimed at facilitating the standardization and harmonization of SARS-CoV-2 antibody tests. The VITROS Immunodiagnostic Products Anti-SARS-CoV-2 IgG Quantitative test was performed using the VITROS Anti-SARS-CoV-2 IgG Quantitative Reagent Pack and the VITROS Anti-SARS-CoV-2 IgG Quantitative Calibrators on the VITROS. Patient sample results were evaluated with a numerical result in Binding Antibody Units (BAU) per mL and with a Nonreactive (negative) or Reactive (positive) label using the above-mentioned cut-off of 17.8 BAU/mL. At the same time, the upper limit of the measuring interval was 4000 BAU/mL. Given the immune deficit caused by MM itself, along with induction therapy prior to ASCT, patients with antibody response greater than 500 BAU/mL were arbitrarily defined as high-responders. Peripheral blood lymphocyte immunophenotyping by flow cytometry was used, when available, to identify the B-lymphocyte CD19+ antibody-producing population. All patients were programmed for general and COVID-19-specific humoral immunity evaluation before mobilization, transplantation, and in follow-up visits one, three, and six months following ASCT, when available in our center. Patients scheduled for double ASCT were evaluated twice if anti-SARS-CoV-2 IgG antibodies remained greater than 17.8 BAU/mL before the second ASCT.

### 2.2. Statistical Analysis

A total of 40 MM patients with confirmed COVID-19 vaccine response (anti-SARS-CoV-2 IgG quantitative test greater than 17.8 BAU/mL) that underwent ASCT were initially programmed for antibody response monitoring. However, due to the change in the anti-SARS-CoV-2 IgG quantitative test used at our center, the study was closed before completing enrollment, causing a significant impact on both data consistency and the study’s statistical power. Summary statistics were used to describe patients’ characteristics for both the continuous and categorical variables. Numerical variables evaluated were age, anti-SARS-CoV IgG antibody response, and CD19+ B-lymphocyte count at different time points, as per protocol. Categorical variables, on the other hand, were as follows: gender (male, female), monoclonal protein type, number of vaccine doses prior to ASCT (one, two, three), immunoglobulin levels IgG (cut-off 700 mg/dL), IgA (70 mg/dL), IgM (40 mg/dL), induction treatment type. Normal distribution of baseline anti-COVID-19 antibody level prior to ASCT was confirmed using Shapiro–Wilk test (*p* = 0.3). Friedman test was used to analyze the eventual progressive impact in terms of SARS-CoV-2 antibody level reduction following ASCT compared to baseline, and a two-tailed *p* value < 0.05 was considered to be statistically significant.

All calculations were performed using MedCalc version 12.30.0.0 (Producer: MedCalc Software bvba, Ostend (Belgium) www.medcalc.org, accessed on 15 July 2024).

## 3. Results

### 3.1. Study Population

A total of 14 MM patients with a median age of 55 years (range 34–67 years) and confirmed AR (targeted antibody titer level greater than 17.8 BAU/mL) underwent 19 ASCT reinfusions and were monitored between January and December 2022. Five patients received tandem autologous transplants and were therefore evaluated for 3 months following the first ASCT and re-evaluated starting from the second ASCT, given the maintenance of detectable SARS-CoV-2 antibody response. Most patients were treated in first-line treatment (71%) using a bortezomib–thalidomide–dexamethasone (VTD) regimen. One patient alone received ASCT following second-line daratumumab–lenalidomide–dexamethasone (DaraRD) treatment. On the other hand, in the rest of the study population bortezomib–cyclophosphamide–dexamethasone was used in two patients (one had both MM and AL amyloidosis), while another one was treated using daratumumab–bortezomib–cyclophosphamide–dexamethasone as part of a clinical trial in first-line. Three patients achieved complete response (CR) before ASCT (21%); very good partial response (VGPR) was present in 4 (29%), while 7 (50%) had a partial response (PR). Cyclophosphamide was most commonly used as a mobilization regimen at a dosage of 2 mg/m^2^ i.v. in all but one patient, who received growth cell-stimulating factors.

The median period from the last vaccination dose to ASCT reinfusion was 14 weeks (range 2–28 weeks). Six patients (43%) received the two-dose mRNA regimen prior to the first ASCT, and the third “booster” dose following the second ASCT, while all three doses were administered before transplantation in half of the study population. One patient received a two-dose mRNA regimen prior to the second ASCT, while another one had documented paucisymptomatic COVID-19 infection and received only one vaccine dose, as per local regulatory policy. The other three patients suffered from a mild form of COVID-19 infection after ASCT. All patients received mRNA vaccines, and NT162b2 was used in most of them. Patient characteristics and vaccination status at baseline are described in Table 1.

### 3.2. Autologous Stem Cell Transplantation and SARS-CoV-2 Antibody Response Monitoring

All patients received a high-dose melphalan conditioning regimen prior to ASCT reinfusion, at 200 mg/m^2^ full dosage in 12 (63%), while a reduced 140 mg/m^2^ dose was used for comorbidity status in 7 of them. The median stem cell infusion dose was 4.72 × 10^6^/kg (range 2.7–13).

The median anti-SARS-CoV-2 IgG baseline level was 311 BAU/mL before nineteen ASCT reinfusions (ranging from 20 to 4000 BAU/mL). The impact of cyclophosphamide mobilization on AR was evaluated in eleven episodes, with the persistence of extremely high-level antibody titer in four cases, comparing antibody level prior to mobilization chemotherapy and prior to ASCT reinfusion (>4000 BAU/mL) independently from CR/VGPR status, while a median 39% reduction was present in seven of them.

Median follow-up was three months after ASCT (range 1–9). Antibody response levels at baseline, one, three, and six months following ASCT are described in Table 2, evidencing a significant median drop of 40% of antibody level following the first three months to a median AR of 188 BAU/mL.

However, in nine patients evaluated six months following the last ASCT, the AR rose to a median level of 435 BAU/mL, mainly due to the third “booster” dose and paucisymptomatic COVID-19 infections following the second stem cell transplantation. Anti-COVID-19 levels at baseline and during follow-up, along with loss of immune response, are described in Table 2.

In six patients who received the third booster dose three months following the last ASCT, three of them (50%) had a median AR increase of 391 BAU/mL (range 56–1280). Three patients had COVID-19 infection following ASCT, and all of them presented with a mild clinical form. The anti-COVID-19 antibody levels were 311 BAU/mL, 151 BAU/mL, and 53 BAU/mL, respectively one, three, and nine months after ASCT and before the infection, and they were all treated successfully in an outpatient manner.

The 53-year-old patient with both MM and AL amyloidosis experienced pneumonia-related death not associated with SARS-CoV-2 three weeks following ASCT and was not included in AR monitoring follow-up. It should be mentioned that the patient was in partial response and had high Eastern Cooperative Oncology Group (ECOG) performance status prior to ASCT due to progressive development of motor deficit of all four limbs and percutaneous endoscopic gastrostomy-based alimentation. As for the others, they maintained a very good partial or complete response and remained alive at the last follow-up while receiving maintenance therapy.

Interestingly low IgA (<70 mg/dL) and IgM levels (<40 mg/dL) were observed in 7 (39%) and 10 (55%) out of 18 ASCT episodes with documented hypogammaglobulinemia, while the number further reduced at six months to 33% each in 9 evaluated patients, probably due to immune reconstitution. Five patients underwent peripheral blood lymphocyte immunophenotyping by flow cytometry, in three and two patients, respectively, following the first and second ASCT. Three patients received a standard two-dose mRNA regimen prior to flow cytometry, while the third dose was also administrated in the other two prior to ASCT. There was a mean increase of 73/mm^3^ CD19+ B-lymphocytes count three months after ASCT reinfusion compared to baseline (range 19–159/mm^3^) and maintenance of CD4+ T-lymphocyte count greater than 200/mm^3^ in four of them at three months. The antibody response was present in all of them, with anti-COVID-19 levels between 135 and 351 BAU/mL in three patients and >4000 BAU/mL in two cases. Two of the above-mentioned patients received the third “booster” dose three months after ASCT, and both of them maintained AR greater than 500 BAU/mL, respectively, 618 BAU/mL and 1750 BAU/mL.

### 3.3. Impact of Third “Booster” Dose and Paucisymptomatic COVID-19 Infection

Patients with AR greater than 500 BAU/mL were defined as high-responders, and while 42% of the study population had higher values at baseline, the number dropped to 39% and 22%, respectively, one and three months after ASCT. The progressive reduction of AR in the first three months compared to baseline was confirmed to be statistically significant using the Friedman test (*p* = 0.014), demonstrating the negative impact of high-dose chemotherapy followed by ASCT. However, both the third dose and mild COVID-19 infection exposure led to an increase of high-responders to 55% (5 out of 9 patients) after six months and a loss of statistically significant negative impact of ASCT (*p* = 0.597), evidencing the positive impact of re-vaccination.

## 4. Discussion

The anti-SARS-CoV-2 antibody response following vaccination has been studied in different populations following the COVID-19 pandemic, including the general population without significant health problems and cancer patients, both in follow-up and during active treatment. The prospective Italian VAX4FRAIL study evaluated SARS-CoV-2 vaccination response in 378 patients that had solid tumors, hematological malignancies, neurological disorders, and immune-rheumatological diseases. The study demonstrated an overall seroconversion rate of 69%, significantly worse compared to healthcare workers. In addition, only 52% of hematological patients had humoral responses with a significantly lower AR compared to oncological and neurological patients [20]. In Australia, the SerOzNET study observed antibody response in 395 adult cancer patients following up to five doses of COVID-19 vaccines, mainly mRNA. Antibody response following a three-dose regimen was achieved in 84% of the study population, while in one-third of the study population with hematological malignancy, only 64% of them had detectable anti-SARS-CoV-2 IgG antibodies and were generally associated with the use of anti-B-cell therapies [21].

Finally, one of the first studies including 67 patients with hematological malignancies alone observed only 54% of COVID-19 seroconversion following a two-dose mRNA vaccine regimen, and B-lymphoproliferative disorders were at particularly high risk of non-response [22].

As for patients that received stem cell transplantation, a retrospective German experience described 243 hematological patients that underwent SARS-CoV-2 vaccination, mainly mRNA in 85%, following allogeneic SCT, achieving seroconversion in 70% of the study population after a median of 2 years from transplantation [23].

Additionally, the French study, including 687 consecutive allogeneic SCT recipients, evidenced a 78% seroconversion rate and was especially efficient when administered at least one year after reinfusion [24]. A recent study from Asimakopoulos and colleagues studied 54 hematological patients who underwent HSCT, mainly autologous in lymphoma and MM, and received the BNT162b2 mRNA vaccine from 6 months before and within 5 years after transplantation without previously documented COVID-19 infection. A total of 86% of the study population achieved AR 3 months after vaccination, and there was a better response rate especially in case of vaccination after at least 18 months from HSCT; however, it was not consistently statistically significant, especially in patients with hypogammaglobulinemia with deficit of IgA [25]. However, there is limited data regarding the long-term impact of chemotherapy in hematological patients with established humoral response. Therefore, we decided to monitor anti-SARS-CoV-2 AR in MM patients following ASCT, evaluating potential antibody titer loss, response to third “booster” dose, and clinical outcome of eventual COVID-19 infections.

A total of 14 MM patients with confirmed AR underwent nineteen ASCT reinfusions (5 patients received tandem ASCT) and were monitored using an Anti-SARS-CoV-2 IgG Quantitative test prior to mobilization, at transplantation time, and in follow-up visits after one, three, and six months. Five patients that received tandem ASCT and maintained AR were evaluated twice. Half of the study population received at least a two-dose mRNA regimen prior to the first ASCT. At the same time, the rest of them underwent three doses prior to transplant, excluding one patient with a single vaccination that suffered from mild/asymptomatic COVID-19 infection (Table 1). All patients achieved at least a partial response prior to ASCT and were subsequently treated with cyclophosphamide mobilization and a high-dose melphalan conditioning regimen before stem cell reinfusion.

Interestingly, the anti-SARS-CoV IgG median baseline level was 311 BAU/mL prior to ASCT, with a negative impact of cyclophosphamide mobilization in terms of AR in around one-third of the study population, and a 40% reduction of the antibody titer (Table 2). However, 20% of them maintained an extremely high response with a titer greater than 4000 BAU/mL both prior to mobilization and the conditioning regimen. The antibody response suffered from a significant drop in the first three months following ASCT in the study population, as expected, from 311 to 188 BAU/mL. The ASCT impact on AR reduction in the first three months following high-dose chemotherapy was confirmed statistically using the Friedman test (*p* = 0.014) and in terms of the number high-responders with an anti-SARS-CoV-2 IgG antibody level greater than 500 BAU/mL that was nearly twice less three months after ASCT compared to baseline (22% and 42%, respectively). The progressive antibody reduction in ASCT patients following mRNA vaccination was consistent with the experience from the Greek study, although maintaining detectable anti-SARS-CoV-2 IgG levels [25]. However, these patients were mainly vaccinated following ASCT and, therefore, could not estimate both the negative impact on already achieved AR and the effect of a potential third “booster” vaccine dose.

Surprisingly, in our study, the antibody level increased to a median level of 435 BAU/mL in nine patients six months after the second ASCT, mainly due to the third “booster” vaccination dose (50% response rate) or SARS-CoV-2 episode, neutralizing the negative impact of chemotherapy associated with the loss of statistically significant difference compared to baseline values (*p* = 0.597). The high-responder population also increased from 22% at three months to 55% (5/9 patients) at six months. The limited number of patients evaluated six months after stem cell infusion was caused by a second ASCT in five patients, three months after the first one, and were therefore re-evaluated from baseline. The positive impact of the third dose in terms of AR was already demonstrated in the Italian VAX4FRAIL study, including hematological patients, although to a lesser extent, with one-third of the non-responders to a standard two-dose regimen that achieved seroconversion. Also, ongoing immunosuppressive treatment and its effect on the immune system prior to vaccination were identified as the most impactful factors of impaired humoral response, and such patients were included in our study population [20]. As for the allogeneic SCT setting in the German retrospective study, factors that were individualized in multivariate analysis were age at the time of allo-SCT, ongoing immunosuppressive therapy, and lack of immune reconstitution in terms of CD4+ T cell count (<200/mm^3^) [23]. The response was independent of the time interval between allo-SCT and vaccination. Furthermore, the impact of the third dose was again confirmed, in both the German and French studies, with the achievement of detectable AR in initial non-responders in over 50% and 41%, respectively [23,24].

Finally, improvement of antibody response in patients with “weak” response (<250 BAU/mL) in the French study was observed in 85% of the above-mentioned patients [24].

It should also be highlighted that none of the three patients who suffered from COVID-19 infection following ASCT had AR greater than 500 BAU/mL but did not need specific inpatient treatment due to mild disease presentation. This could be explained by the impact of the BNT162b2 mRNA vaccine single-dose in healthy donors in terms of both humoral response with the development of anti-RBD and anti-spike antibodies with Fc-mediated effector functions, and cellular response dominated by the CD4+ T cell component [26]. Furthermore, five patients that were monitored using peripheral blood lymphocyte immunophenotyping had an increase in CD19+ B-lymphocytes three months after ASCT reinfusion, maintaining extremely high AR greater than 4000 BAU/mL in two of them. In contrast, the rest had anti-COVID-19 between 135 and 351 BAU/mL. Additionally, most of the patients had CD4+ T-cell counts greater than 200/mm^3^, confirming immune reconstitution, in line with other studies that demonstrated no effect on T-cell response following vaccination in fragile hematological patients during immunosuppressive treatment [20]. The potential mechanism of specific SARS-CoV-2 cellular response, even in the case of impairment of the humoral one, could be due to the essential role of interferon-gamma, resulting in low symptomatic clinical manifestations [27]. Even though the AR can be delayed in immunocompromised patients, as mentioned above, the cellular response, both alone and through cell interaction, reactivates the humoral immunity through memory B-lymphocytes, when available [27].

Our study has major limitations, including a small study population and shorter follow-up than scheduled, that led to incomplete immune reconstitution. Technical issues forced premature study closure before completing enrollment. Based on national policy, mRNA vaccines were used in immunocompromised patients, given the risk of virus reactivation in vaccines based on viral vector; therefore, no data on ChAdOx1-nCoV-19 with adenovirus vector is present in our study population [28].

Finally, based on the phase 3 PROVENT study, the prophylaxis with tixagevimab–cilgavimab has improved the approach in immunocompromised patients, guaranteeing a better outcome and reduced mortality including real-life cohorts [29,30,31].

However, although specific monoclonal antibodies have a duration of at least 6 months in all patients, it is passive prophylaxis and depends on known variants, and after their elimination, there is no specific patient immunity, neither B- nor T-lymphocyte, unlike in the case of active vaccine protection.

## 5. Conclusions

Our study confirms the negative impact of high-dose chemotherapy followed by ASCT on anti-COVID-19 humoral response after anti-SARS-CoV-2 mRNA vaccination with a significant loss of AR in MM patients. However, the administration of the third vaccine dose between three and six months following ASCT improved humoral response in the majority of patients, and the reduced, but still present antibody level was sufficient to prevent severe COVID-19 infection in three patients. These data can be of aid for both the necessity of tixagevimab–cilgavimab use in COVID-19 vaccinated patients with adequate humoral response prior to immunosuppressive treatment, and to further consolidate the timing of re-vaccination not later than six months following ASCT. More studies with larger cohorts and in different settings of patients, both oncological and hematological, including not only chemotherapy but also immune and target therapy along with their impact, are needed to extend the knowledge in terms of maintenance of humoral response and sufficient levels of AR in order to prevent life-threatening COVID-19 infections.

## Figures and Tables

**Table 1:** Study patients’ characteristics, vaccination, and SARS-Co2 infection status.

Category	Study Population
MM patients	14
Median age in years (range)	55 (34–67)
Female, N (%)	4 (29%)
Male, N (%)	10 (71%)
Monoclonal protein type	IgG kappa—4 (29%) IgG lambda—3 (21%) IgA kappa/lambda—3 (21%) Free light chain kappa—4 (29%)
Hypogammaglobulinemia baseline	IgG levels < 700 mg/dL—6 (43%) IgA levels < 70 mg/dL—6 (43%) IgM levels < 40 mg/dL—9 (64%)
Previous treatments, N (%)	VTD—10 (71%) VCD—2 (15%) DaraVCD (trial)—1 (7%) VTD → DaraRD—1 (7%)
mRNA COVID-19 vaccine doses baseline, N (%)	One dose ^1^—1 (7%) Two doses—6 (43%) Three doses—7 (50%)
SARS-CoV-2 infections baseline, N (%)	Paucisymptomatic—4 (29%) No infection—10 (71%)

^1^—Prior SARS-CoV-2 infection. Abbreviations: DaraRD—daratumumab, lenalidomide, dexamethasone, MM—multiple myeloma; VCD—bortezomib, cyclophosphamide, dexamethasone; VTD—bortezomib, thalidomide, dexamethasone.

**Table 2:** Median anti-COVID-19 antibody level at baseline and following ASCT and loss of immune response during follow-up in 19 episodes of ASCT reinfusion.

Category	Study Population
ASCT reinfusions	19 * (five tandem ASCT)
Median anti-COVID-19 antibody level	Baseline—311 BAU/mL One month—276 BAU/mL Three months—188 BAU/mL Six months—435 BAU/mL (8 ASCT)
Median anti-COVID-19 antibody level reduction following cyclophosphamide mobilization	39% (7 ASCT) (range 16–66%) No reduction in 4 ASCT (>4000 BAU/mL)
Anti-COVID-19 antibody level > 500 BAU/mL, N (%)	Baseline—8 (42%) One month—7 (39%) (18 ASCT) Three months—4 (22%) (18 ASCT) Six months—5 (55%) (9 ASCT)
Median anti-COVID-19 antibody level reduction compared to baseline (%)	One month—28% (10 ASCT) Three months—72% (12 ASCT) Six months—61% (4 ASCT)

Abbreviations: ASCT—autologous stem cell transplantation. *—One patient died from pneumonia-related death not associated with SARS-CoV-2 immediately after ASCT and was not included in AR monitoring; therefore, 18 patients were monitored.

## Data Availability

The original contributions presented in this study are included in the article. Further inquiries can be directed to the corresponding author.

## Abbreviations

The following abbreviations are used in this manuscript:

MM	Multiple Myeloma
ASCT	Autologous Stem Cell Transplantations
AR	Antibody Response
SARS-CoV-2	Severe Acute Respiratory Syndrome Coronavirus 2
HSCT	Hematopoietic Stem Cell Transplantation
ECIL	European Conference on Infections in Leukemia
HD-PAM	High-Dose Melphalan
RBD	Receptor-Binding Domain
BAU	Binding Antibody Units
CR	Complete Response
VGPR	Very Good Partial Response
PR	Partial Response
